# Kanglaite Injection Combined with Chemotherapy versus Chemotherapy Alone for the Improvement of Clinical Efficacy and Immune Function in Patients with Advanced Non-Small-Cell Lung Cancer: A Systematic Review and Meta-Analysis

**DOI:** 10.1155/2020/8586596

**Published:** 2020-01-27

**Authors:** Jianxia Wen, Tao Yang, Jian Wang, Xiao Ma, Yuling Tong, Yanling Zhao

**Affiliations:** ^1^Department of Pharmacy, The Fifth Medical Center of PLA General Hospital, Beijing 100039, China; ^2^College of Pharmacy, Chengdu University of Traditional Chinese Medicine, Chengdu 611137, China; ^3^College of Clinical Medicine, Chengdu University of Traditional Chinese Medicine, Chengdu 610075, China

## Abstract

Recent advances have shown that immune checkpoint inhibitors are emerging as promising therapeutic targets to improve the quality of life in cancer patients. This meta-analysis was conducted to evaluate the influence of Kanglaite injection (KLTi) combined with chemotherapy versus chemotherapy alone on clinical efficacy, immune function, and safety for the treatment of advanced non-small-cell lung cancer (NSCLC). Several electronic databases, including PubMed, Web of Science, Wan-Fang, VMIS, EMBASE, Cochrane Library, CNKI, CBM, and MEDLINE, as well as grey literatures, were comprehensively searched from January 2000 to November 2019. Randomized controlled trials (RCTs) reporting outcomes of clinical efficacy and immune function were collected according to their inclusion and exclusion criteria. Cochrane Reviewers' Handbook 5.2 was applied to assess the risk of bias of included trials. STATA 13.0 and Review Manager 5.3 software were used for meta-analysis. Twenty-five RCTs comprising 2151 patients meeting the inclusion criteria were identified. Meta-analysis showed that compared with chemotherapy alone, KLTi plus the same chemotherapy significantly improved clinical efficacy, including complete response, partial response, stable disease, and progressive disease, as well as immune function, including CD^3+^, CD^4+^, CD^8+^, and CD^4+^/CD^8+^. There was a significant reduction in nausea and vomiting, thrombocytopenia, and leukopenia in combination treatments. However, the outcomes were limited because of the low quality and small sample size of the included studies. In conclusion, this work might provide beneficial evidence of KLTi combined with chemotherapy for improving clinical efficacy and immune function, as well as reducing the incidence of adverse events in advanced NSCLC patients. KLTi might be a beneficial therapeutic method for the treatment of advanced NSCLC. Due to the quality of the data, more rigorous and well-designed RCTs are needed to confirm these findings.

## 1. Introduction

Lung cancer remains one of the most common leading causes of cancer-related death, with high incidence rates all over the world [1–3]. Histologically, approximately 80% of these lung cancers are of the non-small-cell type, including squamous cell carcinoma, adenocarcinomas, adenosquamous carcinoma, large cell carcinoma, and sarcomatoid carcinoma [4]. Clinically, advanced non-small-cell lung cancer (NSCLC) accounts for approximately 85% of all cases of lung cancer [5], and the 5-year overall survival rate for patients with metastatic NSCLC was less than 5% [6]. Recently, cisplatin-based chemotherapy was recommended as a first-line treatment for patients with advanced NSCLC in the clinic [7]. Cisplatin-based chemotherapy, such as cisplatin plus vinorelbine, gemcitabine, docetaxel, and pemetrexed, serves as the primary treatment for advanced NSCLC. Carboplatin is a major therapeutic treatment for chemotherapy regiments in patients with comorbidities or in patients not able to tolerate cisplatin. According to clinical trials, these chemotherapies are available for relieving symptoms and prolonging survival in patients with advanced-stage NSCLC [8]. However, these treatments are also limited to a certain extent. Clinically, the effectiveness of chemotherapy alone is not completely satisfactory due to the potential side effects and adverse reactions that affect the quality of life (QOL) and seriously inhibit the immune function of patients [9]. Hence, drugs that exhibit clinical efficacy and promote immune function, improve QOL, and alleviate side effects and adverse reactions may be preferable for advanced NSCLC patients. As one of the most meaningful challenges in drug discovery, more effective and rational drugs for advanced NSCLC remain to be developed.

Recently, traditional Chinese medicine (TCM) combined with chemotherapy to increase effectiveness, reduce side-effects, and improve QOL has shown its advantages as an adjunct therapy for lung cancer treatment [10]. A series of studies seeking novel anticancer drugs has been triggered by the experience-based herbal medicine as a supplementary to modern western medicine [11]. Kanglaite injection (KLTi), an acetone extract of Semen Coicis Yokuinin, is prepared as an herbal medicine using modern and advanced pharmaceutical technology [12]. Notably, KLTi (Zhejiang Kanglaite Group Co. Ltd., Hangzhou, China) is an agent that was approved by the China Food and Drug Administration (CFDA) in 2010. Clinically, KLTi has synergistic effects with radiotherapy and chemotherapy and clearly exerts antievil pathogenic and analgesic effects in advanced lung cancer [13]. The clinical mechanisms of KLTi for advanced NSCLC are related to the induction of cancer cell apoptosis, inhibition of cancer cell mitosis, execution of cancer cells, and improvement of the immune function [14]. Several published systematic reviews and meta-analyses demonstrated that KLTi combined with chemotherapy improves clinical efficacy, performance status, and Karnofsky (KPS) score and reduces radiotherapy and chemotherapy side effects compared with chemotherapy alone in patients with advanced NSCLC [15, 16]. Nevertheless, the improvements in immune function, including peripheral blood T lymphocyte subsets and peripheral blood immunoglobulins, in response to KLTi have not been reported.

**Figure 1:**
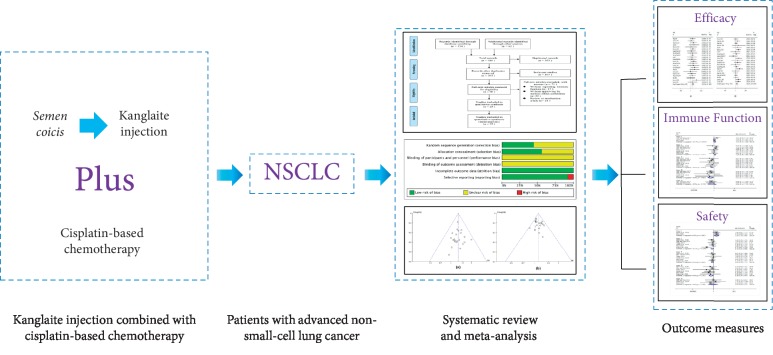
Research strategy of the current study.

Based on previous clinical studies, we performed a systematic review and meta-analysis of KLTi combined with standard chemotherapy in patients with advanced NSCLC. The study objectives were to assess the clinical efficacy, immune function (including CD^3+^, CD^4+^, CD^8+^, CD^4+^/CD^8+^, natural killer (NK) cell count, IgA, IgG, and IgM), adverse events such as nausea and vomiting, thrombocytopenia, and leukopenia of combination therapy in patients with advanced NSCLC (Figure 1). This work could provide comprehensive evidence for further studies on KLTi. In addition, the overall number of KLTi clinical studies on cancer needs to be improved, so that the clinical efficacy and safety of KLTi can be approved by the international community and possibly enter the international market.

## 2. Materials and Methods

### 2.1. Study Registration

This systematic review and meta-analysis had been registered in PROSPERO (https://www.crd.york.ac.uk/PROSPERO/#joinuppage), and the registration number is CRD42018087094.

### 2.2. Ethical Approval and Consent to Participate

As this study does not involve animal and patient experiments, the ethical approval and consent to participate are not applicable.

### 2.3. Literature Source and Search Strategy

PubMed, Web of Science, Wan-Fang database, VIP medicine information system (VMIS), EMBASE, Cochrane Library, China National Knowledge Infrastructure (CNKI) databases, Chinese Biological Medical (CBM), and MEDLINE databases, as well as Baidu and Google, were comprehensively searched from January 2000 to November 2019. In addition to these, grey literatures were searched to identify potential studies that focused on the clinical efficacy and immune function of KLTi combined with chemotherapy in patients with advanced NSCLC. The following search terms were used: (“Kanglaite” [Mesh terms] OR “KLT” [Mesh terms] OR “Kanglaite Injection” [Mesh terms]) AND (“lung cancer” [Mesh terms] OR “lung carcinoma” [Mesh terms] OR “non-small cell lung cancer” [Mesh terms] OR “non-small lung carcinoma” [Mesh terms] OR “NSCLC” [Mesh terms]). Languages were limited to English and Chinese. Search results were downloaded for further evaluation. Furthermore, any additional relevant articles were identified by searching studies included in the reference lists from existing systematic reviews and meta-analyses. We contacted authors whose research reports had questionable data or lacked any relevant information.

### 2.4. Inclusion Criteria

Inclusion criteria were as follows: (1) patients with advanced NSCLC confirmed by cytology or pathology were considered eligible for inclusion in this systematic review; (2) study type included randomized controlled trials (RCTs); and (3) intervention included a control group given routine chemotherapy, including cisplatin plus vinorelbine, gemcitabine, docetaxel, and pemetrexed, while the experimental group was additionally given KLTi with chemotherapy. RCTs with one or more outcomes were included. Citations were screened at the title and abstract level and were subsequently retrieved as full reports.

### 2.5. Exclusion Criteria

Exclusion criteria were as follows: (1) patients could not be confirmed as having advanced NSCLC; (2) neither RCT nor “random” was mentioned in trials; (3) control measures did not include chemotherapy; (4) duplication of previous publications; (5) study was mechanistic, case report, comment, conference abstracts, review, meta-analyses, or letters to the journal editor; and (6) trials with unavailable or incorrect data for meta-analysis.

### 2.6. Observation Index

In this systematic review and meta-analysis, the observation indexes were as follows: (1) evaluation of clinical efficacy rate and improvement of immune function and QOL were performed according to *Guidance for Clinical Research on New Drugs of TCM* [17]. (2) Clinical efficacy of chemotherapy was evaluated in accordance with the *Response Evaluation Criteria in Solid Tumors* (RECIST) developed by World Health Organization (WHO) curative effect evaluation criteria. Patients were divided into four categories: complete response (CR), partial response (PR), stable disease (SD), and progressive disease (PD). The clinical effective rate was considered as the primary endpoint. The disease control rate was characterized as the objective response and stabilization rates. Clinical effective rate and disease control rate were calculated as follows: clinical effective rate = (CR + PR)/total cases × 100%, disease control rate = (CR + PR + SD)/total cases × 100% [18]. (3) Immune function indicators, including peripheral blood T lymphocyte subsets (CD^3+^, CD^4+^, CD^8+^, and CD^4+^/CD^8+^ levels), peripheral blood immunoglobulins (IgA, IgG, and IgM levels), and NK cell counts were pooled for meta-analysis; (4) QOL was assessed using the Functional Assessment of Cancer Therapy-Lung (FACT-L) [19] or the European Organization for Research and Treatment of Cancer (EORTC) QLQC30 [20]. In addition, KPS score was also used to evaluate the QOL; and (5) adverse events or adverse drug reactions (ADEs or ADRs), including incidence of toxic and side effects/adverse reactions before and after treatment, were classified into 0∼IV levels, according to the National Cancer Institute Common Toxicity Criteria version 4.0 (CTC4.0) [21].

### 2.7. Data Extraction

According to the inclusion and exclusion criteria, two investigators independently selected studies after reading the title, abstract, and full-text of the studies. Any relevant disagreements were resolved in consultation with Prof. Zhao. From each included study, the following basic information was independently extracted by two assessors: author's name, year published, sample size, age, gender, TNM stage, KPS score, immune function index, and histologically type, including squamous carcinoma (SQC), adenocarcinomas, adenosquamous carcinoma (ADC), and large-cell carcinomas.

### 2.8. Risk of Bias in Individual Studies

Two investigators assessed the risk of bias for all studies using the Cochrane risk of bias assessment [22]. Quality assessment of each trial was performed by Review Manager 5.3 according to the Cochrane Handbook for Systematic Reviews of Interventions, Version 5.2. Both pairs of investigators reviewed each study independently. For all the relevant outcomes, the quality of each trial was classified using a nominal scale: “Yes” (low risk of bias), “No” (high risk of bias), or “Unclear” (unclear risk of bias). Disagreements were resolved in consultation with Prof. Zhao.

### 2.9. Data Synthesis

Statistical analysis was performed using STATA software (version 13.0, TX, USA) and Review Manager 5.3 software (Cochrane Collaboration, Oxford, UK). We calculated the risk ratio (RR) with 95% confidence interval (95% CI) for dichotomous variables and mean difference (MD) with 95% CI for continuous outcomes. Heterogeneity was evaluated by the magnitude of Tau^2^, Chi^2^, corresponding *p* value, and *I*^2^ statistic. Based on the Mantel–Haenszel (MH) or inverse variance (IV) statistical approach in combination with the data, a fixed effect model was performed with minor heterogeneity when *I*^2^ value was below 50%. Potential heterogeneity was found when the *I*^2^ value was above 50%. First, the sources of heterogeneity were explored. Second, a sensitivity analysis was conducted to detect the robustness of the result, and subgroup analysis was performed due to different stages in each index. Finally, a random effect model was applied to pool data. A funnel plot was used for assessing potential publication bias.

## 3. Results

### 3.1. Study Selection

**Figure 2:**
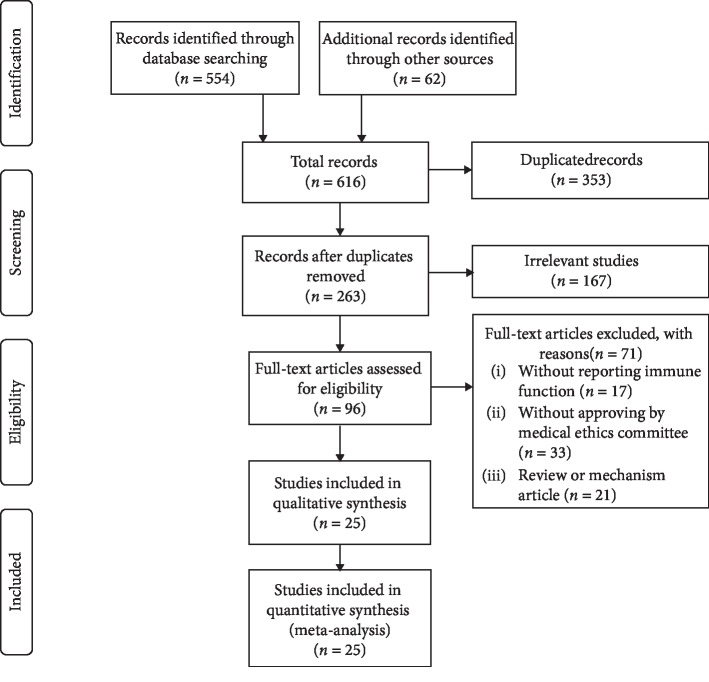
PRISMA flow diagram of study inclusion for this systematic review and meta-analysis.

According to the PRISMA flow diagram, the selection process of eligible studies is illustrated in Figure 2. Five hundred and fifty-four records (554) were identified through database searches, and another 62 records were included from other sources (grey literatures). Following the removal of 353 duplicates, 263 records were included. Next, 167 relevant studies were further removed. Full-text articles of 96 publications were assessed for eligibility. Among them, trials not reporting immune function or approval by a medical ethics committee, as well as those that were a review or mechanistic study, were excluded. Ultimately, 25 studies [8, 23–46] involving 2151 patients (experimental groups: 1103 cases; control groups: 1048 cases) who met the inclusion criteria were included, and the data were extracted from these trials and used for the qualitative analysis.

### 3.2. Study Characteristics

A summary of the baseline characteristics for the included studies is presented in Tables 1 and 2. All these studies were RCTs. Sex differences in advanced NSCLC trends reflect historical differences in tobacco use. Patients' age ranged from 29 to 87 years. Histologically, types of advanced NSCLC were divided into squamous cell carcinoma, adenocarcinomas, adenosquamous carcinoma, and large cell carcinomas, and the details of each trial are shown in Table 2. Twenty-two trials [8, 23–33, 35–37, 40–46] reported outcomes of clinical efficacy, eighteen trials [8, 23–31, 35, 37, 40, 42–46] showed outcomes of adverse reactions, and fifteen trials [8, 25–27, 29, 33, 36, 38, 40–46] reported improvement of QOL. All the trials reported immune function indicators, including peripheral blood T lymphocyte subsets (CD^3+^, CD^4+^, CD^8+^, and CD^4+^/CD^8+^ levels) or/and peripheral blood immunoglobulins (IgA, IgG, and IgM levels) or NK cell counts.

### 3.3. Risk of Bias of Included Trials

**Figure 3:**
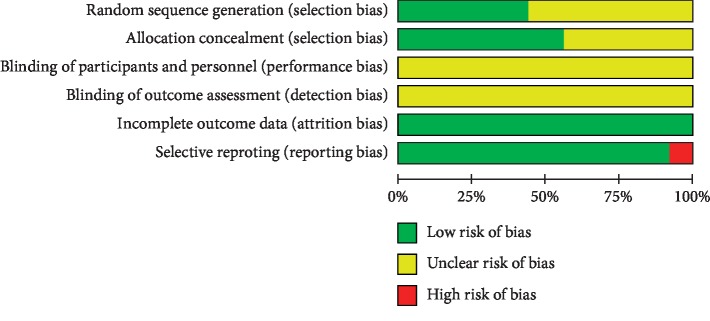
Quality assessment was performed using Review Manager 5.3 according to the Cochrane Handbook for Systematic Reviews of Interventions, Version 5.2. The red square indicates a high risk of bias. The green square indicates a low risk of bias, and the blank square indicates an unclear risk of bias.

The risk of bias for each included trial was evaluated according to the Cochrane risk of bias estimation. All included trials mentioned “randomization,” among which only eight trials [8, 24, 27, 28, 32, 35, 36, 38] stated the appropriate generation with random number table, one trial [33] with stratified randomization method, and two trials [44, 45] with random design paper bags. No trials described information on allocation concealment, blinding of participants and personnel, blinding of outcome assessment, or incomplete outcome data. Selective reporting was performed in two-record [23, 34] (Figure 3).

### 3.4. Clinical Efficacy of KLTi Combined with Chemotherapy

**Figure 4:**
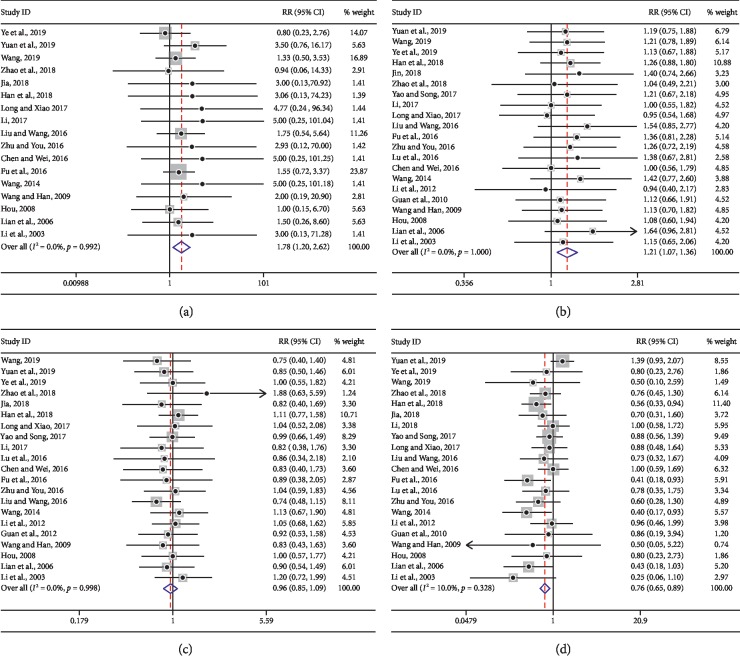
Forest plot of clinical efficacy in advanced NSCLC patients treated with KLTi combined with chemotherapy and chemotherapy alone. (a) CR; (b) PR; (c) SD; and (d) PD.

Clinical efficacy of tumor response was reported in twenty-two trials [8, 23–33, 35–37, 40–46]. As shown in Figure 4, CR, PR, SD, and PD were analyzed in the forms of subgroup. The value of *I*^2^ was less than 50%, indicating that heterogeneity across different trials was not significant (*p* > 0.05), and the fixed effect model was applied in this pooled analysis. Overall, the results of the meta-analysis demonstrated a favorable RR for KLTi treatment CR (RR = 1.78, 95% CI 1.20–2.62) (Figure 4(a)), PR (RR = 1.21, 95% CI 1.07–1.36) (Figure 4(b)), SD (RR = 0.96, 95% CI 0.85–1.09) (Figure 4(c)), and PD (RR = 0.76, 95% CI 0.65–0.89) (Figure 4(d)). It seems that patients in the KLTi group had more CR (60 in the KLTi group vs. 31 in the control group) and PR (378 in the KLTi group vs. 307 in the control group), while those in the control group had more SD (322 in the control group vs. 331 in the KLTi group) and PD (206 in the control group vs. 267 in the KLTi group). These findings indicated that KLTi in combination with chemotherapy exhibited better clinical efficacy than chemotherapy alone.

**Figure 5:**
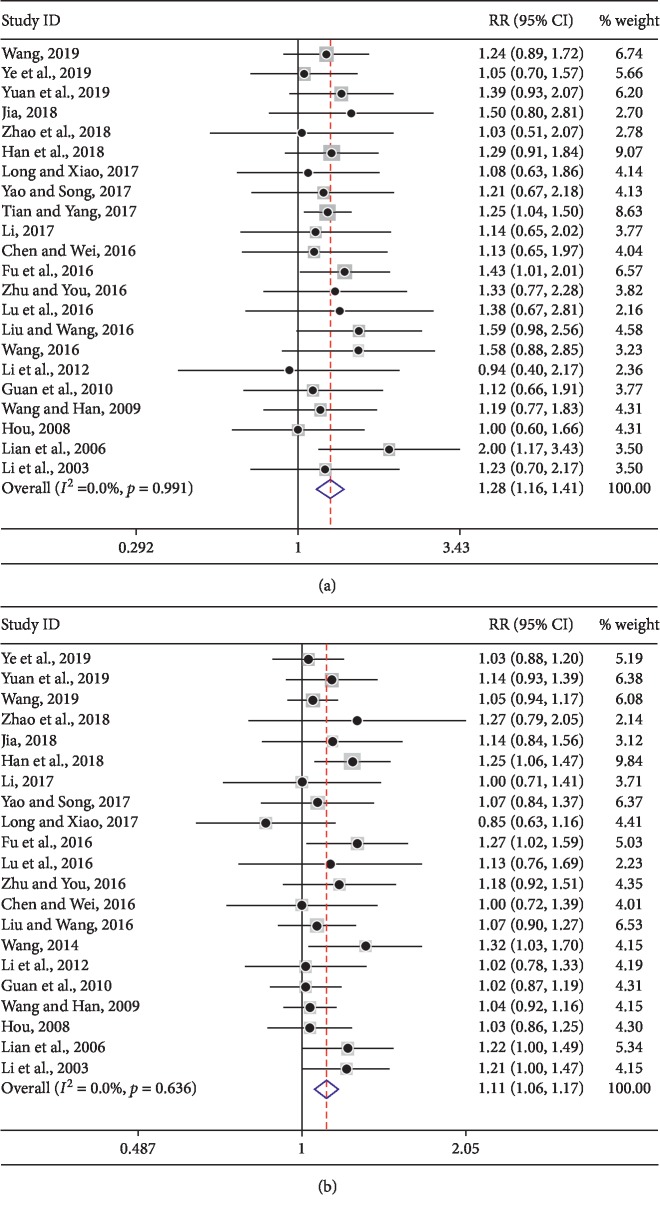
Forest plot of KLTi plus chemotherapy versus chemotherapy alone on (a) short-term clinical effective rate and (b) disease control rate.

Short-term clinical effective rate (CR + PR) and disease control rate (CR + PR + SD) were also evaluated in this study. In the KLTi in combination with the chemotherapy group, 478 of 987 (48.43%) patients reached CR or PR, while 368 of 972 (37.86%) trials achieved CR or PR in the chemotherapy alone group. Our results indicated that KLTi combined with chemotherapy exhibited a superior short-term clinical effective rate (CR + PR) (RR = 1.28, 95% CI 1.16–1.41) (Figure 5(a)) and disease control rate (RR = 1.11, 95% CI 1.06–1.17) (Figure 5(b)) than chemotherapy alone.

### 3.5. Effect of KLTi on Immune Function

Clinical study revealed that KLTi has biphasic antitumor effects. It protects the body's immune system by preventing cancer cell proliferation and transfer and effectively improves immunity in patients with advanced NSCLC [42]. Immune function indicators, including peripheral blood T lymphocyte subsets (CD^3+^, CD^4+^, CD^8+^, and CD^4+^/CD^8+^ levels), peripheral blood immunoglobulins (IgA, IgG, and IgM levels), and NK cell counts were analyzed in this systematic review and meta-analysis.

#### 3.5.1. Peripheral Blood T Lymphocyte Subsets

All studies [8, 23–46] included in this systematic analysis reported data on the percentage of cytotoxic T lymphocytes. As statistical heterogeneity (*p* < 0.00001, *I*^2^ > 50%) was detected in this data, a random effect model was applied for this meta-analysis (Table 3). Results demonstrated that KLTi combined with chemotherapy significantly improved the levels of CD^3+^ (MD = 8.58, 95% CI 6.13–11.04), CD^4+^ (MD = 6.38, 95% CI 4.93–7.83), CD^8+^ (MD = 1.50, 95% CI −0.31–3.32), CD^4+^/CD^8+^ (MD = 0.32, 95% CI 0.25–0.39), and NK cells count (MD = 10.58, 95% CI 7.27–13.90) in NSCLC patients.

#### 3.5.2. Peripheral Blood Immunoglobulins

Five studies [32–35, 39] also evaluated the effect of KLTi on IgA, IgG, and IgM (Table 3). The MD for IgA, IgG, and IgM was as follows: MD = 0.22, 95% CI 0.08–0.35; MD = 1.69, 95% CI: 1.17–2.22; and MD = 0.18, 95% CI 0.09–0.27, respectively (Table 3).

### 3.6. Improvement of KLTi on QOL Score

**Figure 6:**
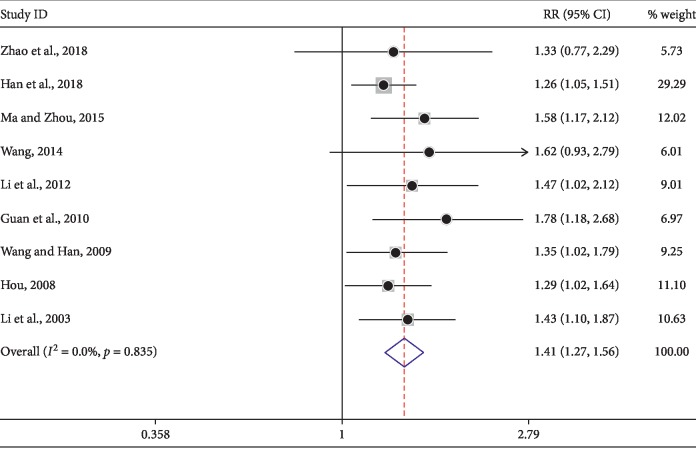
Forest plot of KPS improvement in patients treated with KLTi combined with chemotherapy and chemotherapy alone.

One trial used FACT-L [33] and one trial used QLQC30 [29] to assess the QOL. In addition, KPS score was used in eleven trials [25–27, 38, 40–46] to evaluate the QOL of KLTi on the treatment of NSCLC. Among them, continuous outcomes were used in two trials [25, 45]. No significant heterogeneity (*I*^2^ = 0%, *p*=0.835) was found among another nine trials. Pooled RR revealed that KLTi combined with chemotherapy significantly enhanced KPS improvement compared with chemotherapy alone (RR = 1.41, 95% CI 1.27–1.56) (Figure 6).

### 3.7. Reduction of Adverse Reactions

**Figure 7:**
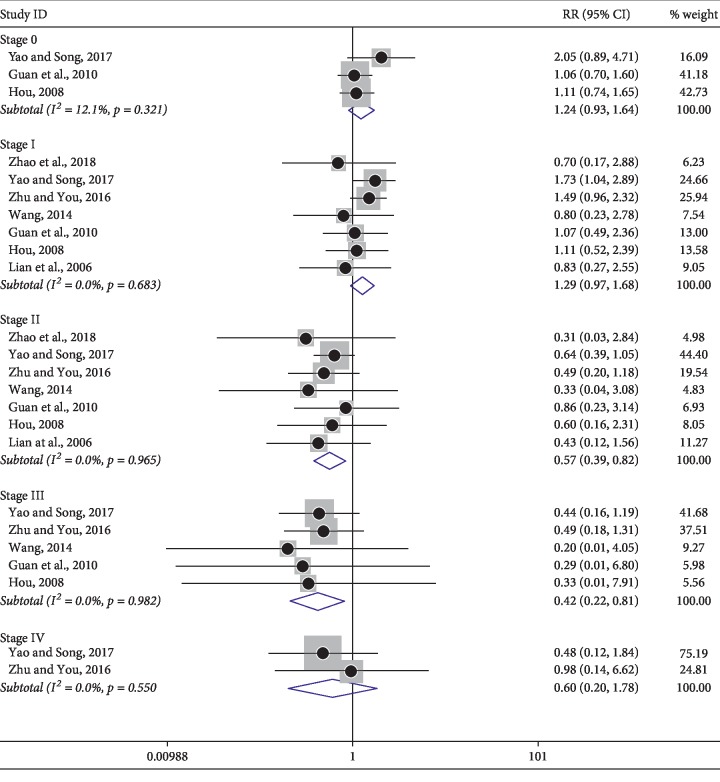
Subgroup analysis of nausea and vomiting in patients with advanced NSCLC treated with KLTi combined with chemotherapy and chemotherapy alone.

**Figure 8:**
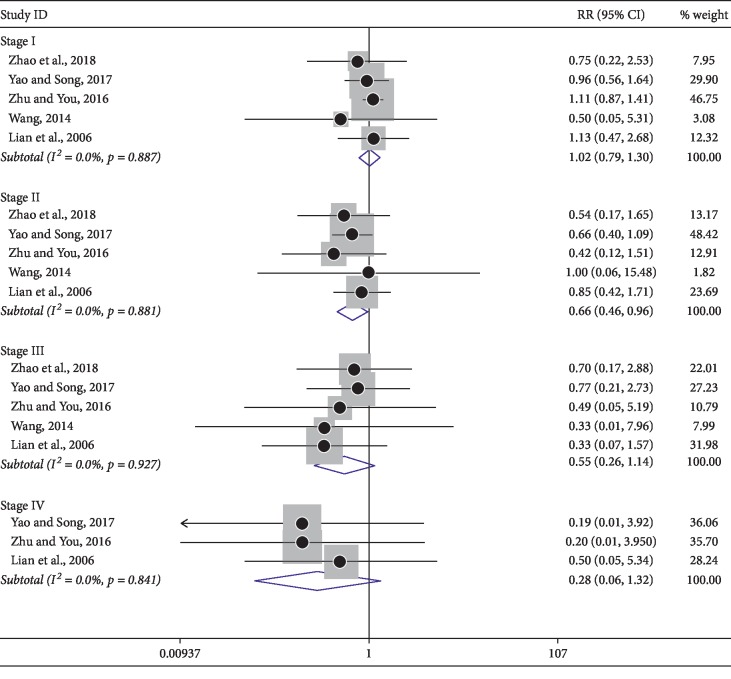
Subgroup analysis of thrombocytopenia in patients with advanced NSCLC treated with KLTi combined with chemotherapy and chemotherapy alone.

**Figure 9:**
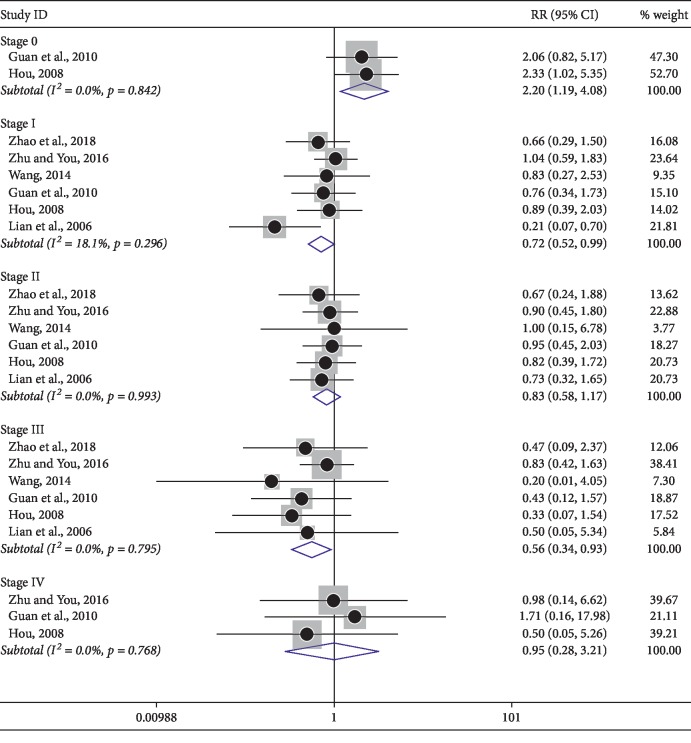
Subgroup analysis of leukopenia in patients with advanced NSCLC treated with KLTi combined with chemotherapy and chemotherapy alone.

Among the included trials, a number of studies analyzed toxicities and side effects/adverse reactions. Notably, patients were divided into stage 0∼IV levels according to the WHO grading criteria for adverse drug reactions. Thus, subgroup analysis among nausea and vomiting (Figure 7), thrombocytopenia (Figure 8), and leukopenia (Figure 9) were carried out based on the same criteria. However, several trials were not divided into 0∼IV levels, so dichotomous measures were used for meta-analysis. As shown in Table 4, a fixed effect model was used for the outcomes. In summary, neither of the two groups experienced serious toxicity or side effects/adverse reactions during treatment. The incidence of adverse events in the treatment group was significantly lower than in control groups, indicating the use of KLTi combined with chemotherapy in patients with advanced NSCLC is superior to chemotherapy alone.

### 3.8. Publication Bias

**Figure 10:**
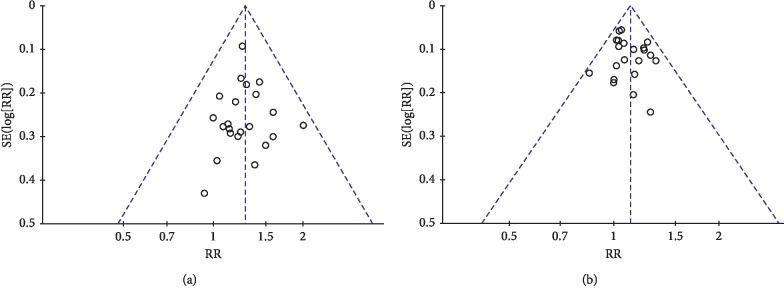
Funnel plot for publication bias of (a) the clinical efficacy and (b) disease control rate.

A funnel plot was created to evaluate publication bias. There were twenty-two trials [8, 23–33, 35–37, 40–46] included in the funnel plot for the clinical efficacy (Figure 10(a)) and twenty-one trials [8, 23–29, 31–33, 35–37, 40–46] included for disease control rate (Figure 10(b)). The results indicated that there was no significant asymmetry observed.

## 4. Discussion

### 4.1. Summary of Evidence

Chemotherapy is a primarily effective therapeutic treatment and has been widely used in routine advanced NSCLC treatment. Numerous studies have indicated that TCM in combination with chemotherapy could be used to increase clinical efficacy and reduce adverse reactions as well as complications from these therapies [47]. KLTi is a botanically sourced and molecularly targeted agent. The patent certificates of KLTi were granted in China in 1995. Notably, phase III clinical trials using KLTi were completed in August 1997. Subsequently, KLTi was officially launched in China after final approval from the Ministry of Public Health [48, 49], which can be combined with chemical, radiological, or targeted therapies in clinical use to decrease cancer burden, improve QOL of cancer patients, and ameliorate multiple drug resistance of cancers [47]. Also, it has been evaluated for preclinical antitumor effects [50]. In pancreatic cells lines, KLTi downregulated protein expression of Bcl-2, increased Fas gene expression, and increased apoptosis [51]. Additionally, KLTi inhibits tumor necrosis factor-alpha- (TNF-*α*-) mediated epithelial mesenchymal transition (EMT) in colorectal cancer cell lines by inhibiting the NF-κΒ signaling pathway [52]. According to research reports, clinical use of KLTi provides objective evidence for the treatment of lung cancer and significantly reduces the expression of miRNA-21 in patients with advanced lung cancer [53]. In this comprehensive systematic review and meta-analysis, we comprehensively evaluated whether KLTi combined with chemotherapy versus chemotherapy alone benefits patients with advanced NSCLC. In particular, our results demonstrate that KLTi had statistically improved clinical efficacy and immune function while reducing adverse reactions in advanced NSCLC in later-line treatments.

### 4.2. Clinical Impact of KLTi on Advanced NSCLC

In the present study, the results were divided into four responses (CR, PR, SD, and PD) (Figures 4(a)–4(d)). Short-term clinical effective rate and disease control rate (DCR) (Figure 5(b)) indicated superior clinical efficacy of KLTi in combination with chemotherapy for the treatment of advanced NSCLC with no significant heterogeneity, consistent with previous studies [12, 15]. To our knowledge, medicines focusing on improving management of cancer patient perspectives, such as QOL, may make a significant difference. Here, KLTi combined with chemotherapy had improved KPS score (Figure 6), reducing the incidence of adverse reactions in patients with advanced NSCLC (Table 4). Appropriate drug selection is a major challenge in patients with advanced NSCLC, especially those who are complicated with concomitant gastrointestinal system and circulatory diseases. Our study helps to put the available safety data of KLTi into perspective. Clinically, when using chemotherapy to treat advanced NSCLC, clinicians can use chemotherapy in combination with KLTi to potentially optimize these regimens.

### 4.3. The Significance of Exploring Immune Function

Recent advances have shown that immune checkpoint inhibitors are emerging as promising therapeutic targets to improve QOL in patients with advanced NSCLC [54]. Of the examination indexes for diagnosing the development, progression, and prognosis of tumors, detection of T lymphocyte subgroups in peripheral blood is of great significance clinically [55]. Clinical studies have shown that KLTi exerts inhibitory effects on the generation of neovascularization in tumor cells, increasing macrophages, further inducing lymphocytic cytokines and tumor necrosis factor, thereby enhancing the immunity capacity of patients [56]. However, its improvement on immune function in patients with advanced NSCLC has not been systematically reported before. The current study investigated whether KLTi in combination with chemotherapy influences the percentage of peripheral blood T lymphocyte subsets (CD^3+^, CD^4+^, CD^8+^ and CD^4+^/CD^8+^) and peripheral blood immunoglobulins (IgA, IgG and IgM) in patients with advanced NSCLC. Our results indicated that KLTi combined with routine chemotherapy was better than routine treatment used alone for these indexes.

### 4.4. Limitations of the Present Study

This study does have some limitations. First, the small sample sizes of included trials in this study were not sufficient to evaluate clinical efficacy or immune function of combination therapy. As a result, we are not sure whether the impact of KLTi is overestimated or underestimated. Second, with different histology and clinical characteristics in the studies, unbalanced baselines existed. Third, the quality of our analysis is limited by the quality of the underlying data. In the risk of bias section, both blinding of participants and personnel (performance bias) and blinding of outcome assessment (detection bias) were not reported in all trials. In addition, analysis indicated that potential publication bias might influence the results of this systematic review. Furthermore, all included trials were published in Chinese. Notably, different NSCLC patients have different TNM stages, and KLTi may have different therapeutic efficacy and prognosis. In this study, the clinical efficacy and safety of KLTi in the treatment of NSCLC were analyzed with unclear TNM staging. The results of our systematic review must be interpreted with caution. Therefore, we hope our present work provides useful experience on further studies on KLTi. As a consequence of these limitations, we clearly find that the overall level of clinical research needs to be improved, so that the clinical efficacy and safety of TCM can be evaluated by the international community to possibly enter the international market. Therefore, more rigorous and well-designed RCTs are needed to confirm these findings.

## 5. Conclusions

In summary, the present results suggest that KLTi, in combination with chemotherapy, might be an effective regimen for the treatment of advanced NSCLC, but further prospective studies with larger numbers of patients are required to fully establish the clinical efficacy and safety of this treatment.

## Figures and Tables

**Table 1:** Baseline characteristics of included studies.

Studies	Cases (E/C)	Sex (M/F)	Age (years) (range, mean)	Stage of TNM	KPS score
Yuan et al. [23]	60/60	E: 34/26	E: 44∼79, 65.11 ± 5.61	E: III36, IV24	E: 60∼89, 74.11 ± 3.55
		C: 36/24	C: 46∼77, 64.39 ± 5.64	C: III34, IV26	C: 61∼88, 73.34 ± 3.51
Ye et al. [24]	40/40	E: 26/14	E: 55∼74, 61.4 ± 9.27	E: III22, IV18	NR
		C: 28/12	C: 56∼77, 63.1 ± 10.34	C: III21, IV19	
Wang [25]	45/45	E: 24/21	E: 62.05 ± 9.87	E: II11, III22, IV12	>60
		C: 23/22	C: 61.73 ± 9.64	C: II13, III21, IV11	
Han et al. [26]	99/101	E: 62/37	E: 50∼78, 60.58 ± 6.13	E: III48, IV51	NR
		C: 65/36	C: 49∼79, 61.02 ± 6.25	C: III50, IV51	
Zhao et al. [27]	32/30	E: 20/12	E: 65∼72	E: IIIB17, IV15	≥60
		C: 16/14	C: 65∼71	C: IIIB17, IV13	
Jia [28]	31/31	E: 18/13	E: 43∼74, 57.06 ± 6.21	E: IIIB18, IV13	>60
		C: 16/15	C: 44∼72, 58.42 ± 4.33	C: IIIB17, IV14	
Li [8]	41/41	E: 22/19	E: 55∼74, 60.4 ± 10.2	E: IIIA9, IIIB13, IV:19	NR
		C: 21/20	C: 55∼75, 61.3 ± 10.7	C: IIIA10, IIIB12, IV19	
Long and Xiao [29]	42/40	E: 27/15	E: 50∼70, 58.46 ± 8.43	E: IIIB15, IV27	>60
		C: 25/15	C: 47∼69, 58.74 ± 8.63	C: IIIB13, IV27	
Tian and Yang [30]	42/42	E: 26/16	E: 53∼86, 64.39 ± 4.27	NR	NR
		C: 27/15	C: 54∼87, 64.87 ± 4.69		
Yao and Song [31]	70/67	E: 41/29	E: 64.8 ± 7.2	E: IIIB45, IV25	E: 65.6 ± 2.5
		C: 37/30	C: 63.4 ± 7.0	C: IIIB41, IV26	C: 67.2 ± 2.1
Liu and Wang [32]	55/55	E: 30/25	E: 46∼79, 54 ± 5	E: III35, IV20	E: 63∼88, 68 ± 5
		C: 32/23	C: 45∼78, 56 ± 5	C: III36, IV19	C: 61∼89, 68 ± 5
Fu et al. [33]	53/47	E: 33/20	31∼75	E: IIIA13, IIIB29, IV11	E: 67.9 ± 2.2
		C: 30/17		C: III10, IIIB27, IV10	C: 68.2 ± 1.5
Zheng et al. [34]	32/32	E: 20/12	E: 46∼71, 58.61 ± 3.52	NR	NR
		C: 19/13	C: 47∼72, 59.22 ± 3.40		
Zhu and You [35]	43/42	E: 25/18	E: 48∼73, 60.22 ± 3.24	III41, IV44	≥60
		C: 24/18	C: 56∼80, 57.12 ± 3.58		
Chen and Wei [36]	44/44	E: 24/20	E: 55∼78, 60.5 ± 10.5	E: IIIA10, IIIB14, IV20	NR
		C: 23/21	C: 55∼78, 60.6 ± 10.8	C: IIIA11, IIIB13, IV20	
Lu et al. [37]	24/24	28/20	44∼71, 58.7 ± 5.8	IIIB26, IV22	>60
Ma an d Zhou [38]	50/50	60/40	68∼80, 72.34 ± 2.22	NR	NR
Deng et al. [39]	34/34	E: 20/14	E: 63.88 ± 1.99	NR	≥80
		C: 22/12	C: 64.29 ± 2.07		
Wang [40]	43/43	E: 29/14	E: 46∼79, 60.07 ± 5.43	E: III34, IV9	≥70
		C: 29/14	C: 43∼79, 59.79 ± 6.02	C: III33, IV10	
Li et al. [41]	38/40	E: 23/15	E: 70∼77	E: IIIA20, IIIB∼IV18	≥60
		C: 26/14	C: 70∼76	C: IIIA21, IIIB∼IV19	
Guan et al. [42]	35/30	E: 20/15	E: 70∼79	E: IIIB15, IV20	NR
		C: 20/10	C: 70∼82	C: IIIB12, IV18	
Wang and Han [43]	30/30	E: 18/12	E: 35∼75	E: III18, IV12	>60
		C: 17/13	C: 35∼74	C: III18, IV12	
Hou [44]	34/34	E: 20/14	E: 37∼71	E: IIIB18, IV16	≥70
		C: 21/13	C: 35∼68	C: IIIB17, IV17	
Lian et al. [45]	50/50	E: 33/17	E: 30∼70	E: IIIA8, IIIB22, IV20	70∼100
		C: 34/16	C: 29∼72	C: IIIB9, IIIB24, IV17	
Li et al. [46]	36/36	E: 27/9	E: 60∼78, 68.5	NR	>50
		C: 28/8	C: 60∼77, 67.9		

Keys: KLTi, Kanglaite injection; E, experimental group (KLTi plus chemotherapy); C, control group (chemotherapy treatment alone); M: male; F: female; KPS, Karnofsky; NR: not reported.

**Table 2:** Characteristics of included meta-analyses on KLTi in combination with chemotherapy for advanced NSCLC.

Studies	Pathology (SCC/A/others)	Intervention	Clinical efficacy	Adverse reactions	QOL	Immune function
Yuan et al. [23]	E: 19/15/26	E: GP + KLTi (200 mL/d)	✓	✓	NR	CD3^+^, CD4^+^, CD8^+^
	C: 18/21/38	C: GP				
Ye et al. [24]	E: 20/18/2	E: GP + KLTi (200 mL/d)	✓	✓	NR	CD3^+^, CD4^+^, CD8^+^
	C: 20/17/3	C: GP				
Wang [25]	NR	E: NP + KLTi (200 mL/d)	✓	✓	✓	CD3^+^, CD4^+^, CD8^+^, CD4^+^/CD8^+^
		C: NP				
Han et al. [26]	E: 52/44/3	E: GP + KLTi (200 mL/d)	✓	✓	✓	CD3^+^, CD4^+^, CD8^+^, CD4^+^/CD8^+^
	C: 55/43/3	C: GP				
Zhao et al. [27]	E: 16/14/2	E: GP + KLTi (200 mL/d)	✓	✓	✓	CD3^+^, CD4^+^, CD8^+^
	C: 15/13/2	C: GP				
Jia [28]	E: 14/15/2	E: DP + KLTi (200 mg/d)	✓	✓	NR	CD3^+^, CD4^+^, CD8^+^, CD4^+^/CD8^+^
	C: 13/16/2	C: DP				
Li [8]	E: 28/9/4	E: GP + KLTi (100 mL/d)	✓	✓	✓	CD4^+^, CD25^+^
	C: 28/9/4	C: GP				
Long and Xiao, [29]	E: 8/20/14	E: GP + KLTi (200 mL/d)	✓	✓	✓	CD3^+^, CD4^+^
	C: 7/19/14	C: GP				
Tian and Yang, [30]	E: 17/23/2	E: DP + KLTi (200 mg/d)	✓	✓	NR	CD3^+^, CD4^+^, CD8^+^, CD4^+^/CD8^+^
	C: 16/22/4	C: DP				
Yao and Song [31]	E: 34/31/5	E: GP + KLTi (200 mL/d)	✓	✓	NR	CD3^+^, CD4^+^, CD8^+^, CD4^+^/CD8^+^
	C: 32/31/4	C: GP				
Liu and Wang [32]	E: 22/19/14	E: NP + KLTi (200 mL/d)	✓	NR	NR	CD3^+^, CD4^+^, CD8^+^, CD4^+^/CD8^+^, IgA, IgG, IgM
	C: 23/18/14	C: NP				
Fu et al. [33]	E: 19/22/12	E: NP + KLTi (200 mL/d)	✓	NR	✓	CD3^+^, CD4^+^, CD4^+^/CD8^+^, NK, IgA, IgG, IgM
	C: 17/19/11	C: NP				
Zheng et al. [34]	E: 15/17/0	E: DP + KLTi (200 mg/d)	NR	NR	NR	CD3^+^, CD4^+^, CD8^+^, CD4^+^/CD8^+^, IgA, IgG, IgM
	C: 14/18/0	C: DP				
Zhu and You [35]	45/38/2	E: GP + KLTi (200 mL/d)	✓	✓	NR	CD3^+^, CD4^+^, CD8^+^, CD4^+^/CD8^+^, IgA, IgG, IgM
		C: GP				
Chen and Wei [36]	E: 10/29/5	E: GP + KLTi (200 mL/d)	✓	NR	✓	CD3^+^, CD4^+^, CD8^+^
	C: 10/29/5	C: GP				
Lu et al. [37]	20/24/4	E: DP + KLTi (200 mg/d)	✓	✓	NR	CD3^+^, CD4^+^, CD8^+^, CD4^+^/CD8^+^
		C: DP				
Ma and Zhou [38]	NR	E: TP + KLTi (100 mL/d)	NR	NR	✓	CD3^+^, CD4^+^, CD4^+^/CD8^+^, NK
		C: TP				
Deng et al. [39]	17/46/5	E: DP + KLTi (200 mg/d)	NR	NR	NR	CD3^+^, CD4^+^, CD8^+^, CD4^+^/CD8^+^, CD4^+^ CD25^+^, IgA, IgG, IgM
		C: DP				
Wang, [40]	E: 24/18/1	E: GP + KLTi (200 mL/d)	✓	✓	✓	CD3^+^, CD4^+^, CD8^+^, CD4^+^/CD8^+^
	C: 23/18/2	C: GP				
Li et al. [41]	NR	E: TP + KLTi (100 mL/d)	✓	NR	✓	CD3^+^, CD4^+^, CD8^+^, CD4^+^/CD8^+^, NK
		C: TP				
Guan et al. [42]	E: 20/15/0	E: GP + KLTi (200 mL/d)	✓	✓	✓	CD3^+^, CD4^+^, CD8^+^, CD4^+^/CD8^+^
	C: 18/12/0	C: GP				
Wang and Han [43]	E: 10/15/5	E: NP + KLTi (200 mL/d)	✓	✓	✓	CD3^+^, CD4^+^, CD8^+^, CD4^+^/CD8^+^, NK
	C: 13/14/3	C: NP				
Hou, [44]	E: 15/19/0	E: NP + KLTi (100 mL/d)	✓	✓	✓	CD3^+^, CD4^+^, CD8^+^, CD4^+^/CD8^+^
	C: 15/19/0	C: NP				
Lian et al. [45]	E: 25/16/9	E: GP + KLTi (200 mL/d)	✓	✓	✓	CD4^+^/CD8^+^
	C: 27/16/7	C: GP				
Li et al. [46]	NR	E: NP + KLTi (200 mL/d)	✓	✓	✓	CD3^+^, CD4^+^, CD8^+^, CD4^+^/CD8^+^
		C: NP				

Keys: KLTi, Kanglaite injection; E, experimental group; C, control group; SCC, squamous cell carcinoma; A, adenocarcinoma; other pathology includes large cell carcinoma and adenosquamous carcinoma; GP, gemcitabine + platinum; DP, docetaxel + platinum; NP, navelbine + platinum; TP, Taxol + platinum; NK, natural killer cells; NR, not reported; QOL: quality of life.

**Table 3:** Comparison of peripheral blood T lymphocyte subsets and peripheral blood immunoglobulins between the two groups before and after treatment.

Outcome	Studies	Participants	Statistical method	Effect estimate
CD3^+^ T cells	23 [23–44, 46]	2009	Mean difference (IV, random, 95% CI)	8.58 [6.13, 11.04]
CD4^+^ T cells	24 [8, 23–44, 46]	2091	Mean difference (IV, random, 95% CI)	6.38 [4.93, 7.83]
CD8^+^ T cells	20 [23–28, 30–32, 34–37, 39–44, 46]	1727	Mean difference (IV, random, 95% CI)	1.50 [–0.31, 3.32]
CD4^+^/CD8^+^ T cells	19 [25, 26, 28, 30–35, 37–46]	1660	Mean difference (IV, random, 95% CI)	0.32 [0.25, 0.39]
NK cells	4 [33, 38, 41, 43]	338	Mean difference (IV, random, 95% CI)	10.58 [7.27, 13.90]
IgA	5 [32–35, 39]	427	Mean difference (IV, random, 95% CI)	0.22 [0.08, 0.35]
IgG	5 [32–35, 39]	427	Mean difference (IV, random, 95% CI)	1.69 [1.17, 2.22]
IgM	5 [32–35, 39]	427	Mean difference (IV, random, 95% CI)	0.18 [0.09, 0.27]

**Table 4:** Reduction of KLTi on adverse reactions when combined with chemotherapy in patients with advanced NSCLC.

Outcome	Studies	Participants	Statistical method	Effect estimate
Nausea and vomiting	9 [24–26, 28–30, 37, 43, 45]	778	Risk ratio (M-H, fixed, 95% CI)	0.52 [0.44, 0.62]
Liver and kidney dysfunction	5 [26, 28–30, 37]	476	Risk ratio (M-H, fixed, 95% CI)	0.76 [0.48, 1.18]
Thrombocytopenia	5 [25, 26, 28, 30, 37]	484	Risk ratio (M-H, fixed, 95% CI)	0.47 [0.30, 0.75]
Anemia	3 [28, 30, 37]	194	Risk ratio (M-H, fixed, 95% CI)	0.28 [0.16, 0.49]
Granulocytopenia	3 [28, 30, 37]	194	Risk ratio (M-H, fixed, 95% CI)	0.34 [0.23, 0.51]
Leukopenia	4 [25, 26, 43, 46]	422	Risk ratio (M-H, fixed, 95% CI)	1.41 [0.31, 0.54]
